# Thoracolumbar Biomechanical Analysis of Lenke Type 1 Adolescent Idiopathic Scoliosis Across Roussouly Classifications

**DOI:** 10.1002/jsp2.70148

**Published:** 2025-12-01

**Authors:** Zhihua Wu, Huantong Cheng, Jia He, Xiaowei Dai, Junyu He, De Liang, Xiaobing Jiang, Yueli Sun, Ruitao She, Yuanfang Lin, Ziyang Liang, Wei Wei

**Affiliations:** ^1^ The First Clinical Medical College Guangzhou University of Chinese Medicine Guangzhou Guangdong China; ^2^ Department of Pediatrics Nanshan Maternal and Child Health Hospital Shenzhen Guangdong China; ^3^ Innovation and Transformation Center, Rehabilitation Technology Innovation Center by Joint Collaboration of Ministry of Education and Fujian Province, Academy of Integrative Medicine, Fujian Key Laboratory of Integrative Medicine on Geriatrics Fujian University of Traditional Chinese Medicine Fuzhou FuJian China; ^4^ Department of Tuina and Spinal Orthopaedics in Chinese Medicine, Shenzhen Traditional Chinese Medicine Hospital The Fourth Clinical Medical College of Guangzhou University of Chinese Medicine Shenzhen Guangdong China; ^5^ Department of Spinal Surgery First Affiliated Hospital of Guangzhou University of Chinese Medicine Guangzhou Guangdong China; ^6^ Department of Spinal Surgery The Second Affiliated Hospital of Guangzhou Medical University Guangzhou Guangdong China; ^7^ Key Laboratory of the Ministry of Education of Chronic Musculoskeletal Disease Longhua Hospital, Spine Institute, Shanghai University of Traditional Chinese Medicine Shanghai China; ^8^ Laboratoire de Biomécanique Appliquée (UMRT24) Aix‐Marseille Université/Université Gustave Eiffel Marseille France

**Keywords:** adolescent idiopathic scoliosis, biomechanics, finite element, intervertebral disc, Roussouly classification

## Abstract

**Background:**

Lenke type 1 is the most common adolescent idiopathic scoliosis (AIS), and its sagittal morphology critically influences progression and treatment. However, its biomechanical characteristics across Roussouly types remain unclear.

**Purpose:**

To quantify the biomechanical responses of Lenke type 1 AIS under pure bending moments across different Roussouly classifications.

**Methods:**

This study was based on a validated thoracolumbar finite element model. Using mesh morphing, spinal alignments and vertebral rotations were adjusted to construct finite element models of Lenke type 1 AIS with Roussouly types 1–4. Simulations were conducted under ±7.5 Nm pure bending moments for flexion‐extension, lateral bending, and axial rotation. Spinal range of motion (ROM) and intervertebral disc loadings—including force, moment, and Von Mises stress—were quantified.

**Results:**

Compared to the normal model, the AIS model showed asymmetrical total ROM at the T7–T12 segment, whereas the T1–S1 segment remained relatively symmetrical. At the T9–T10 and T12–L1 discs, shear and compressive forces increased markedly, with peak values of 197 N and secondary moments reaching ~2.8 Nm. Stress in the T9–T10 disc exhibited a distinct concave‐side concentration, with the maximum Von Mises stress reaching 7.7 MPa. The T1–S1 ROM during extension, right bending, and right rotation in Roussouly 1 and 2 was ~10% greater than in Roussouly 3 and 4, with markedly higher shear and compressive forces (up to 50‐fold) at the T6–T7 and T9–T10 discs. Regarding stress distribution, Von Mises stress at the T6–T7 and T9–T10 discs was higher in Roussouly 3 and 4, whereas stress at the T12–L1 disc was more pronounced in Roussouly 1 and 2.

**Conclusion:**

The findings underscore the critical role of sagittal morphology in AIS biomechanics. Compared to Roussouly 1 and 2, Roussouly 3 and 4 exhibited reduced ROM, lower disc forces, and more favorable stress distributions, suggesting a biomechanically advantageous load‐bearing pattern.

## Graphical abstract

Finite element models of Lenke type 1 adolescent idiopathic scoliosis were morphed into Roussouly sagittal types 1–4 and subjected to pure moment loading. The figure illustrates how sagittal morphology modulates thoracolumbar biomechanics, with Roussouly types 3–4 showing reduced range of motion and more favorable disc loading than types 1–2.

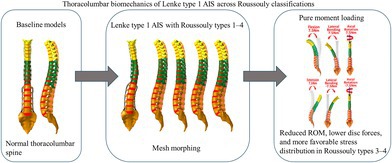

## 1 Introduction

Adolescent idiopathic scoliosis (AIS) is a complex three‐dimensional spinal deformity characterized by multiple vertebrae deviating from the midline in the coronal plane, alterations in kyphosis or lordosis in the sagittal plane, and axial vertebral rotation [1]. Epidemiological data indicate that the global prevalence of AIS may reach 3% and is continuing to rise [2]. Severe AIS may lead to adverse long‐term health consequences, including impaired pulmonary function, disability, chronic back pain, psychological effects, cosmetic concerns, and reduced quality of life [3]. According to the Lenke classification, which primarily focuses on coronal plane deformities, AIS is categorized into six major types, with Lenke type 1 being the most common, accounting for approximately 40% of cases [4, 5].

Relevant studies have demonstrated that sagittal imbalance is closely associated with patients' health‐related quality of life [6, 7]. Compared to coronal imbalance, sagittal imbalance exerts a more profound impact both pre‐ and post‐treatment. Roussouly et al. [8] proposed a classification system based on sacral slope and the apex of lumbar lordosis, dividing the sagittal profile of the normal spine into four types. Roussouly type 1 is characterized by a sacral slope < 35°, small lumbar lordosis, and long thoracolumbar kyphosis. Type 2 also has a sacral slope < 35° and small lumbar lordosis, but presents with a flat‐back profile. Type 3 features a sacral slope of 35°–45° with balanced curvatures. Type 4 is defined by a sacral slope > 45°, long lumbar lordosis, and a short, pronounced thoracic kyphosis. Since being applied to assess sagittal morphology in AIS, the Roussouly classification has been widely recognized for its value in surgical planning by spine surgeons [9, 10, 11]. The use of this classification can reduce the incidence of postoperative mechanical complications, particularly proximal junctional kyphosis, thereby improving clinical outcomes [12, 13, 14]. Although the Roussouly classification has enhanced our understanding of sagittal alignment in AIS, no studies have yet investigated the biomechanical differences across the various curve types. This gap presents a crucial entry point for exploring the relationship between AIS progression mechanisms and spinal kinematics.

Range of motion (ROM) is a key parameter for evaluating spinal flexibility and is likely influenced by different curve types in AIS. Studies have shown that, compared to the normal population, AIS patients do not exhibit reduced mobility in the sagittal and coronal planes [15]. Instead, they demonstrate increased mobility in specific segments above and below the apex, suggesting a compensatory mechanism in AIS spines [15]. This compensation may involve biomechanical adaptations across spinal segments, with sagittal morphology playing a crucial role in spinal movement patterns. Variations in sagittal morphology, as defined by the Roussouly classification, may influence ROM and compensatory strategies. Additionally, the intervertebral disc, as the primary load‐bearing and motion structure of the spine, plays a vital role in AIS development [16]. The study by Schlösser et al. [17] found that torsion, anterior overgrowth, and coronal wedging of the intervertebral discs in AIS patients were at least three times greater than those of the vertebrae. They concluded that, compared to bony structures, intervertebral discs contribute more substantially to the three‐dimensional deformity of AIS. However, due to the challenges of directly measuring intervertebral disc mechanical changes in vivo, studies on its biomechanical behavior primarily rely on finite element (FE) simulations. Reports indicate that, under various loading conditions, lumbar intervertebral discs in AIS patients exhibit reduced resistance to torsional loads, rendering them more susceptible to stress concentrations and structural damage [18]. However, existing FE models often represent individual cases or small cohorts, thereby limiting the generalizability of biomechanical findings. A previous study quantitatively compared the biomechanical responses of normal and AIS spines under pure bending moments [19]. The results indicated that the AIS model exhibited greater ROM asymmetry and intervertebral disc loading variations, with differences observed across various Lenke types. However, that study focused solely on coronal plane factors and did not incorporate sagittal plane parameters, particularly the biomechanical implications of the Roussouly classification.

Therefore, this study aims to investigate spinal biomechanical behavior in AIS through FE simulation, with particular emphasis on how the Roussouly classification—a sagittal alignment system—affects spinal mechanical characteristics. A healthy control model and FE models of Lenke type 1 AIS representing Roussouly types 1–4 will be developed to comprehensively evaluate their effects on spinal ROM and intervertebral disc loadings. This study is expected to offer novel insights into the biomechanical pathogenesis of AIS and serve as a theoretical foundation for personalized treatment strategies.

## 2 Research Method

### 2.1 AIS Thoracolumbar Spine FE Models

The thoracolumbar spine FE model used in this study was based on a previously developed model [19], which was originally extracted from the Total Human Model for Safety pedestrian model to represent the biomechanical characteristics of the high‐risk AIS population. Specifically, the model incorporates key anatomical components of the spine and employs various element types and material properties to accurately simulate the biomechanical behavior of the vertebrae, intervertebral discs, and ligaments. To ensure reliable biomechanical responses, the thoracolumbar FE model was validated in the previous study [19] through material parameter optimization and calibration under dynamic compression and quasi‐static bending moments. Detailed descriptions of the validation procedures, including model configurations and mechanical responses, are provided in the Methods section and supporting information of the referenced study [19].

In this study, the validated thoracolumbar FE model was further utilized to construct Lenke type 1 AIS models corresponding to different Roussouly classifications. The average coronal Cobb angle in Lenke type 1 AIS patients has been reported as 28.85° ± 19.94° [20]. Based on this, the previously developed FE model from our earlier study [19], constructed using a representative X‐ray image with a T7–T12 Cobb angle of 28°, was used as the reference for coronal adjustment. The extracted thoracolumbar FE model underwent coronal alignment adjustment via mesh morphing in HyperMesh (version 2019, Altair Engineering, Troy, MI)—a technique that has been increasingly applied in recent years to generate subject‐specific spinal FE models based on medical imaging data [21, 22]. Mesh morphing offers clear advantages in preserving element quality, anatomical accuracy, and mesh continuity during large‐scale deformations [22]. These features make it particularly suitable for simulating morphological variations such as scoliosis deformities, including changes in curvature and vertebral rotation. This technique has been effectively used to investigate biomechanical differences across spinal subtypes by enabling consistent comparative analyses while maintaining geometric realism [19]. Specifically, manual freehand morphing and mapping functions were used to iteratively translate and rotate each vertebra to adjust disc heights until the final T7–T12 Cobb angle reached 28°.

**FIGURE 1:**
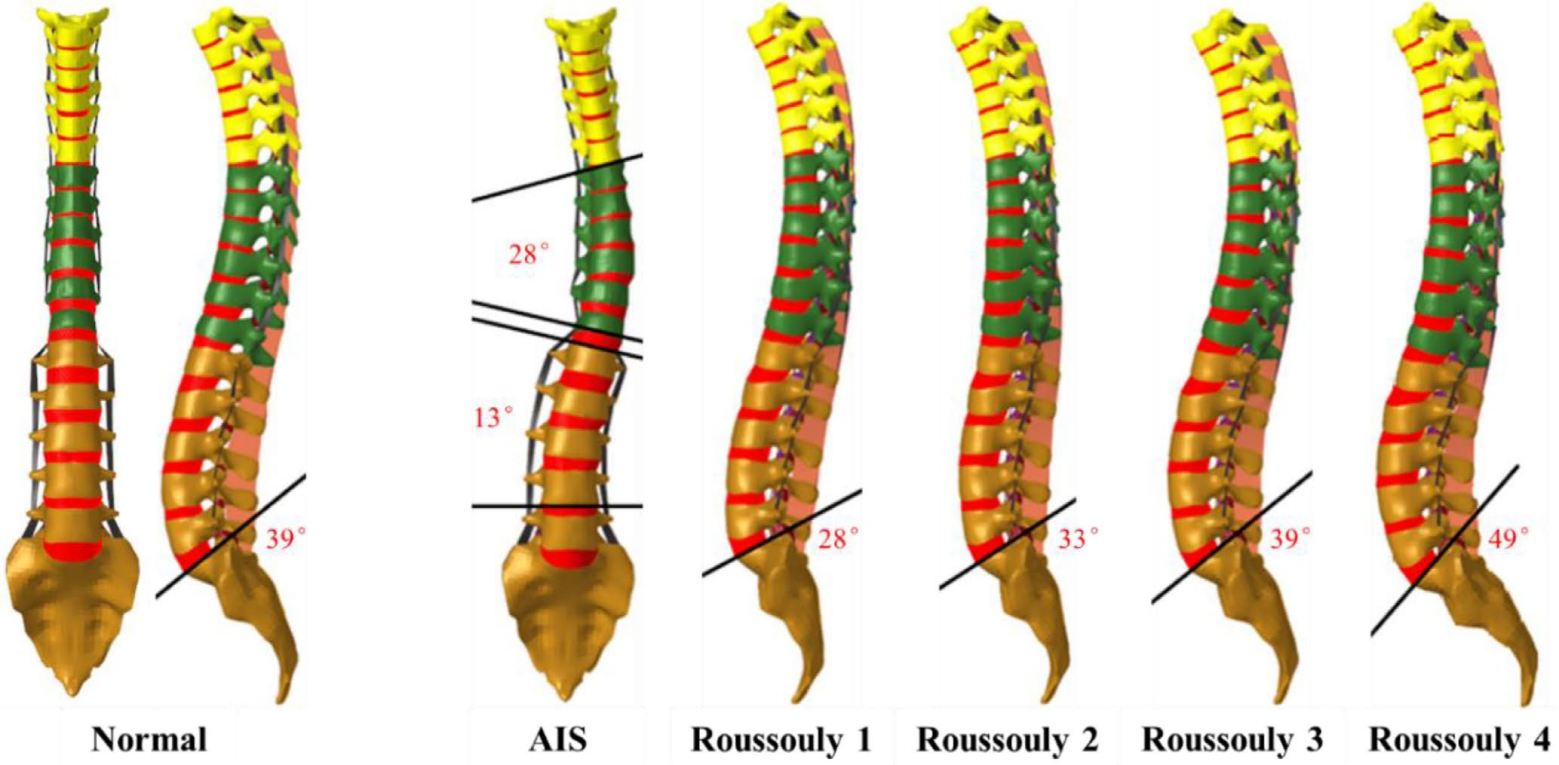
Anterior–posterior and lateral views of each finite element model, including normal and Roussouly 1–4.

After coronal plane adjustment, vertebral axial rotations were further refined according to the average rotation angles of each vertebra reported for Lenke type 1 AIS patients in the published data [23], to ensure conformity with typical axial rotation patterns. The adjusted rotation angles for all vertebrae are summarized in Table A1. Based on this, sagittal curvature was modified according to the four sagittal parameters of the Roussouly classification, including sacral slope and the position of the lumbar lordosis apex, as reported by Hong et al. [20], resulting in the development of FE models for Roussouly types 1 to 4. The sacral slopes for Roussouly 1 to 4 were 28°, 33°, 39°, and 49°, respectively. The original thoracolumbar FE model exhibited a sacral slope of 39°, consistent with Roussouly type 3 characteristics, and had coronal and axial rotation angles close to 0°. To construct a matching normal model, sagittal curvature was adjusted to align with the sagittal profile of Roussouly 3. In total, five FE models were established: Normal and Roussouly 1 to 4. The anterior–posterior and lateral views of each model are presented in Figure 1. The spinal curvature parameters in the sagittal, coronal, and axial planes for all models are provided in Table A1.

### 2.2 Biomechanical Analysis Under Pure Bending Moments

A systematic biomechanical analysis was conducted on five thoracolumbar FE models under pure bending moments to investigate the influence of different sagittal morphologies on spinal kinematics and intervertebral disc loadings. To ensure the reliability of the simulation results and align with relevant experimental studies [24], the sacrum of all models was fully constrained in all degrees of freedom. A pure moment of ±7.5 Nm was applied to the superior end of T1 to simulate six motion modes: flexion, extension, left and right lateral bending, and left and right axial rotation.

To evaluate overall spinal kinematics, the total ROM of the T7–T12 and T1–S1 segments was measured. The T7–T12 segment, as the primary region of scoliotic curvature, reflects the impact of spinal deformity on local mobility, while the T1–S1 segment represents overall thoracolumbar ROM and serves as a key indicator of spinal flexibility and compensatory capacity. Furthermore, to analyze the kinematic characteristics of different spinal segments under various loading conditions, the ROM of the T1–T6, T7–T12, and L1–S1 segments was measured in the coronal, sagittal, and axial planes. T7 and T12 are critical transitional vertebrae, serving as the upper and lower end vertebrae of scoliosis, and located near the transition region where curvature frequently changes direction, effectively dividing the FE model into three segments.

Similarly, the T6–T7 and T12–L1 discs are located near the transitional regions of scoliosis and serve as key sites for stress redistribution. The T9–T10 disc, located at the apex of scoliosis, directly reflects the mechanical characteristics of the peak scoliotic region. Therefore, to investigate the loading patterns of intervertebral discs under different motion states, the bearing forces and moments of the T6–T7, T9–T10, and T12–L1 discs were measured in the coronal, sagittal, and axial planes. Subsequently, to comprehensively evaluate intervertebral disc stress under different conditions, the Von Mises stress on the T6–T7, T9–T10, and T12–L1 discs was analyzed by dividing each disc into four regions: front, rear, left, and right. Stress levels in each region were measured and statistically analyzed to identify stress concentration areas, thereby exploring the load distribution and potential biomechanical adaptation mechanisms.

All FE simulations in this study were performed using the explicit solver in LS‐DYNA 971 R11.1 (LSTC, Livermore, CA, USA) on an Intel Xeon workstation. For statistical analysis, the Roussouly 3 AIS model was compared with Normal, which was derived from the previously validated thoracolumbar FE model of Roussouly type 3 and adjusted to match the sagittal alignment of the Roussouly 3 AIS model. This consistency in sagittal morphology facilitated variable control and enabled a focused comparison of biomechanical responses between AIS patients and typical healthy spines. All statistical analyses were performed using Python 3.12.8 with the SciPy and statsmodels libraries. Pairwise comparisons among the four Roussouly types were conducted using paired t‐tests to identify biomechanical differences, based on anatomically matched regions under identical loading and boundary conditions. This ensured a paired data structure, and the t‐test was considered appropriate given its robustness to minor deviations from normality. Sample sizes were as follows: vertebral ROM—17 paired samples (sacrum excluded); IVD regional stresses—T6–T7 (front 12, left 9, rear 18, right 9), T9–T10 (front 24, left 12, rear 32, right 12), and T12–L1 (front 52, left 22, rear 64, right 22). Statistical significance was set at *p* < 0.05.

## 3 Results

### 3.1 Spinal Range of Motion Analysis

**FIGURE 2:**
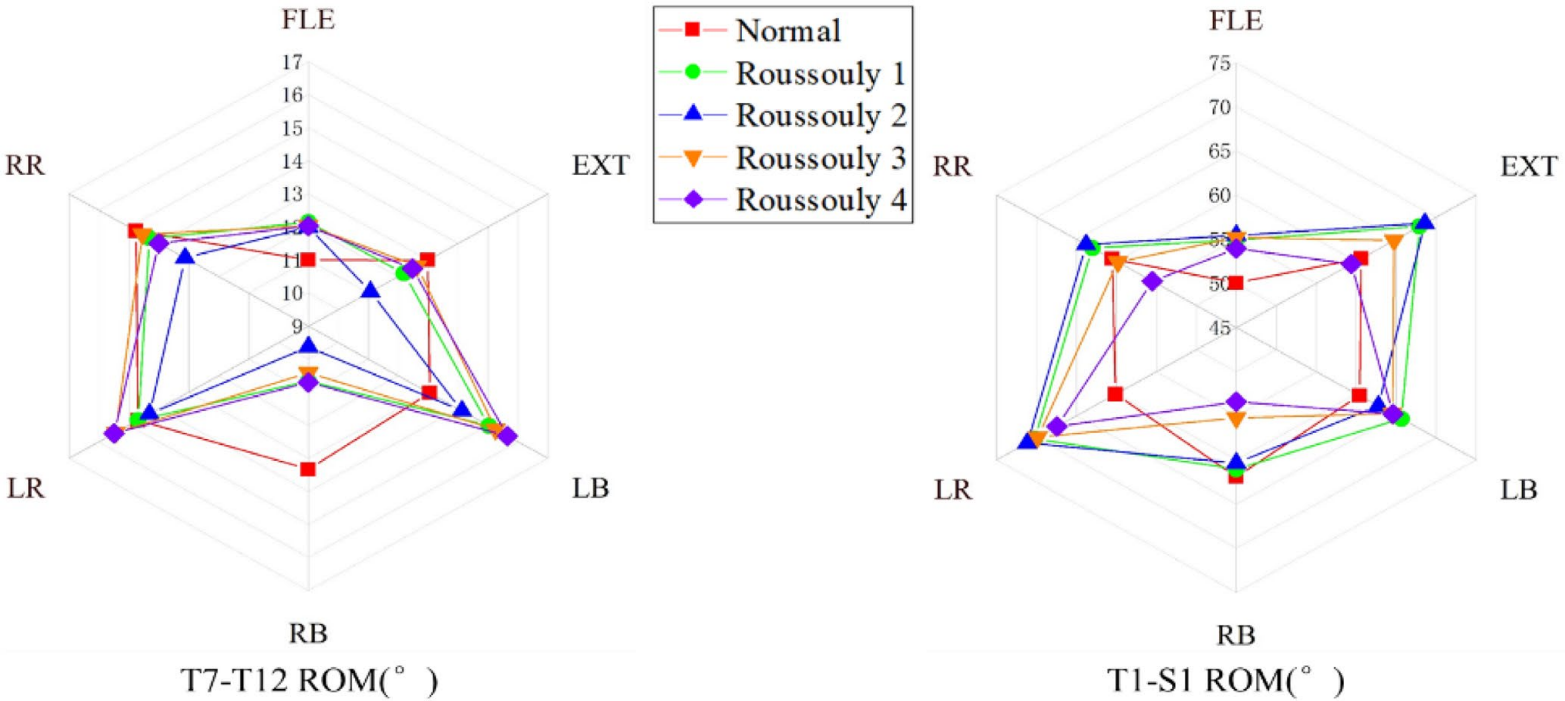
Range of motion (ROM) of the T7–T12 and T1–S1 segments in each model under six loading conditions. EXT, extension; FLE, flexion; LB, left bending; LR, left rotation; RB, right bending; RR, right rotation.

The overall ROM of the T7–T12 and T1–S1 segments for each model is shown in Figure 2. In the T7–T12 segment, all AIS models exhibited a greater ROM than the normal model under flexion and left bending conditions, while their ROM was lower under extension, right bending, and right rotation. Notably, under right bending, the ROM of Roussouly 1–4 was 80.0%, 72.2%, 78.1%, and 80.3% of Normal, respectively. Additionally, the ROM of the AIS models was greater under left bending and left rotation conditions than under right bending and right rotation. Among the AIS models, Roussouly 2 exhibited the smallest ROM in nearly all conditions. In the T1–S1 segment, the AIS models had a greater ROM than the normal model under flexion, left bending, and left rotation conditions, but a smaller ROM under right bending. The ROM variation among different Roussouly classifications generally followed the trend: Roussouly 1 ≈ 2 > 3 > 4.

**FIGURE 3:**
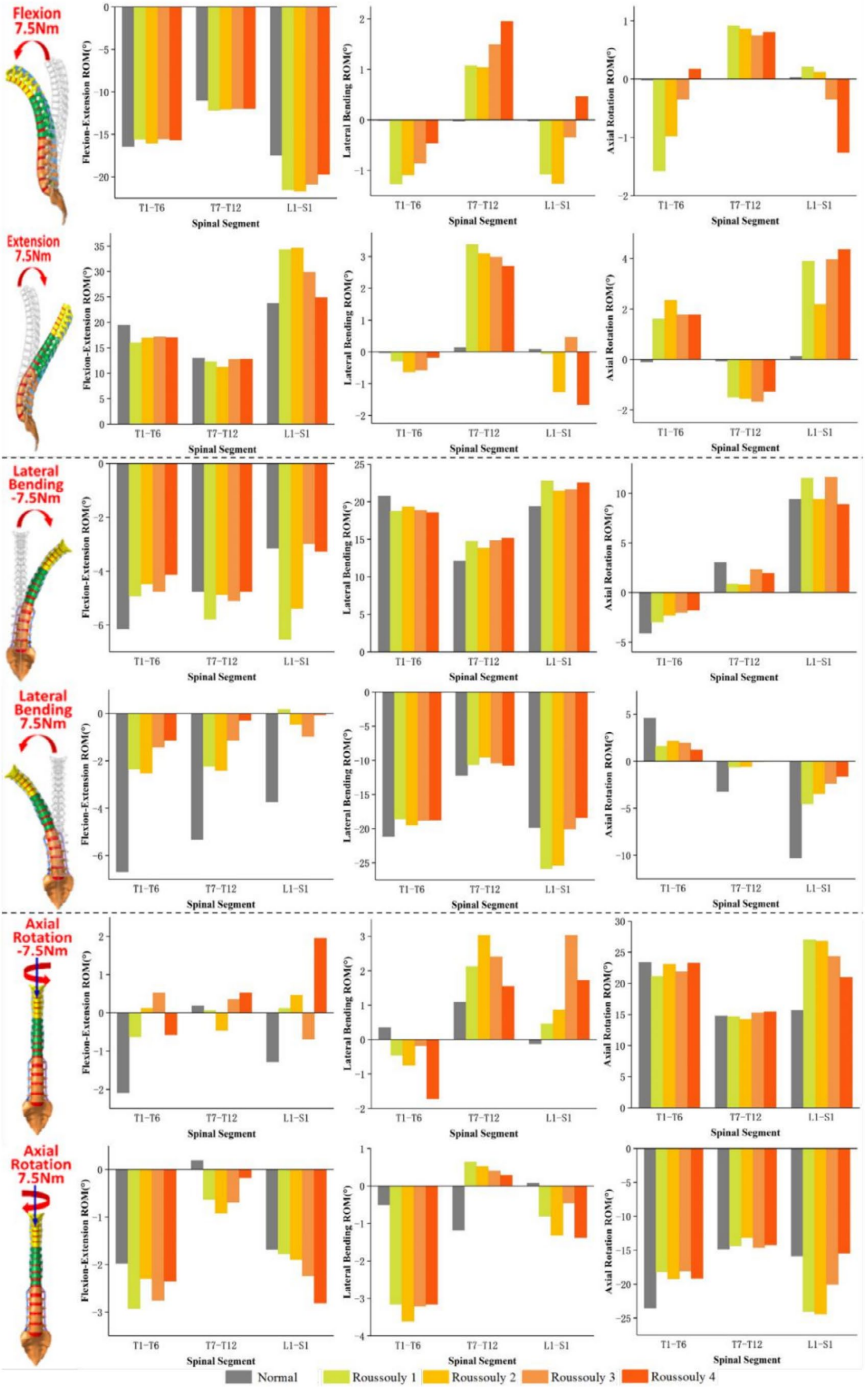
Three‐dimensional range of motion (ROM) of the T1–T6, T7–T12, and L1–S1 segments in each model under six loading conditions.

The ROM of the T1–T6, T7–T12, and L1–S1 segments in the coronal, sagittal, and axial planes for each model is shown in Figure 3. In the primary motions, the ROM of the T1–T6 segment in Roussouly 3 decreases compared to Normal, while it increases in the L1–S1 segment. A comparison among AIS models shows that Roussouly 2 has a relatively larger ROM in the T1–T6 segment but a smaller ROM in the T7–T12 segment. In the L1–S1 segment, the ROM of Roussouly 1 and 2 is generally greater than that of Roussouly 3 and 4. In coupled motions, the highest ROM in the T1–T6 segment almost always occurs in Roussouly 1 or 2, while the lowest (except for rotation) is primarily observed in Roussouly 4. Similarly, in the T7–T12 segment, Roussouly 1 generally exhibits the largest ROM among the AIS models, while Roussouly 4 is relatively smaller. However, in the L1–S1 segment, the ROM of Roussouly 4 is often the largest among the AIS models.

**FIGURE 4:**
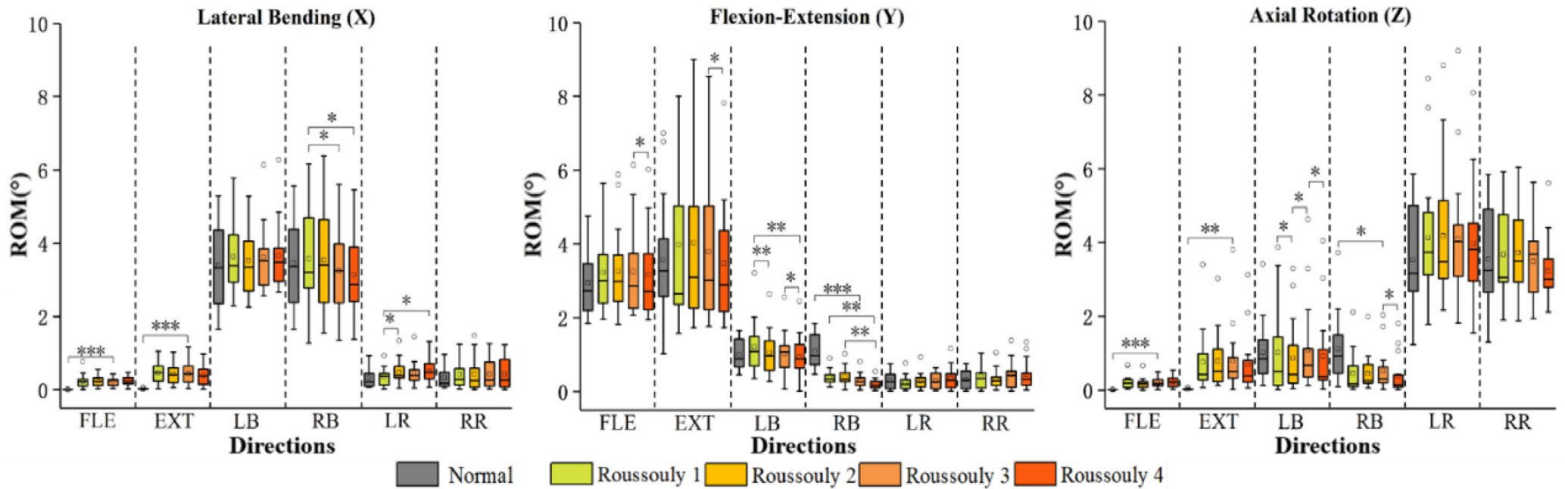
Boxplot of the three‐dimensional range of motion (ROM) for each vertebra in each model under six loading conditions.

Figure 4 illustrates the three‐dimensional distribution of ROM across each vertebra in different models. In primary motions, the ROM of Roussouly 1 is significantly greater than that of Roussouly 3 and 4 during right bending. In coupled motions, during left bending along the Y direction, the ROM of Roussouly 1 is significantly greater than that of Roussouly 2 and 4, while Roussouly 3 also shows a higher ROM than Roussouly 4. Along the Z direction, the ROM of both Roussouly 1 and 3 is significantly greater than that of Roussouly 2, and Roussouly 3 is also significantly higher than Roussouly 4. During right bending, along the Y direction, the ROM of both Roussouly 1 and 2 exceeds that of Roussouly 4, while in the Z direction, Roussouly 3 shows a significantly higher ROM than Roussouly 4.

### 3.2 Intervertebral Disc Mechanical Loading Analysis

**FIGURE 5:**
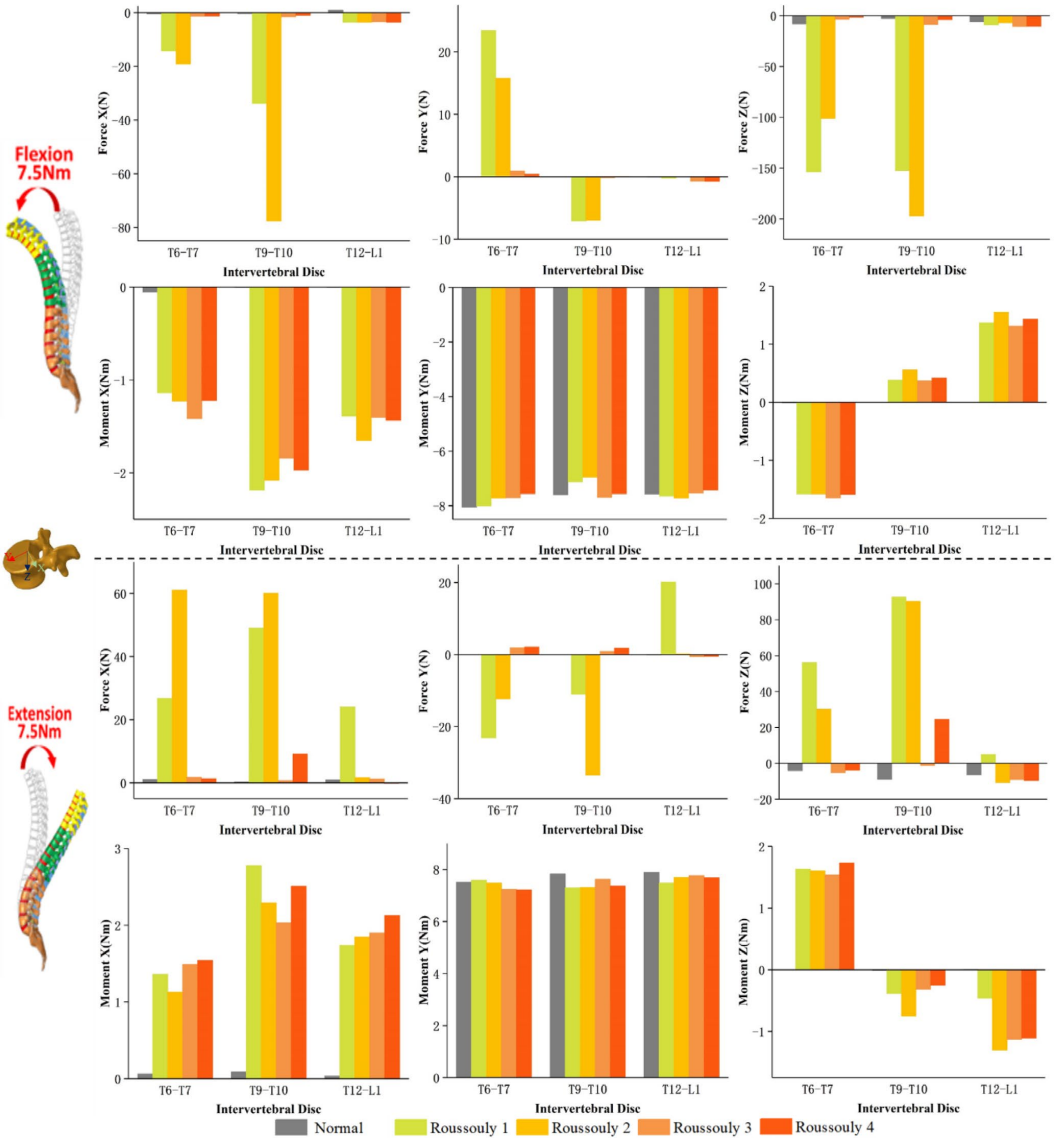
Three–dimensional forces and moments sustained by the T6–T7, T9–T10, and T12–L1 discs in each model under flexion–extension loading.

**FIGURE 6:**
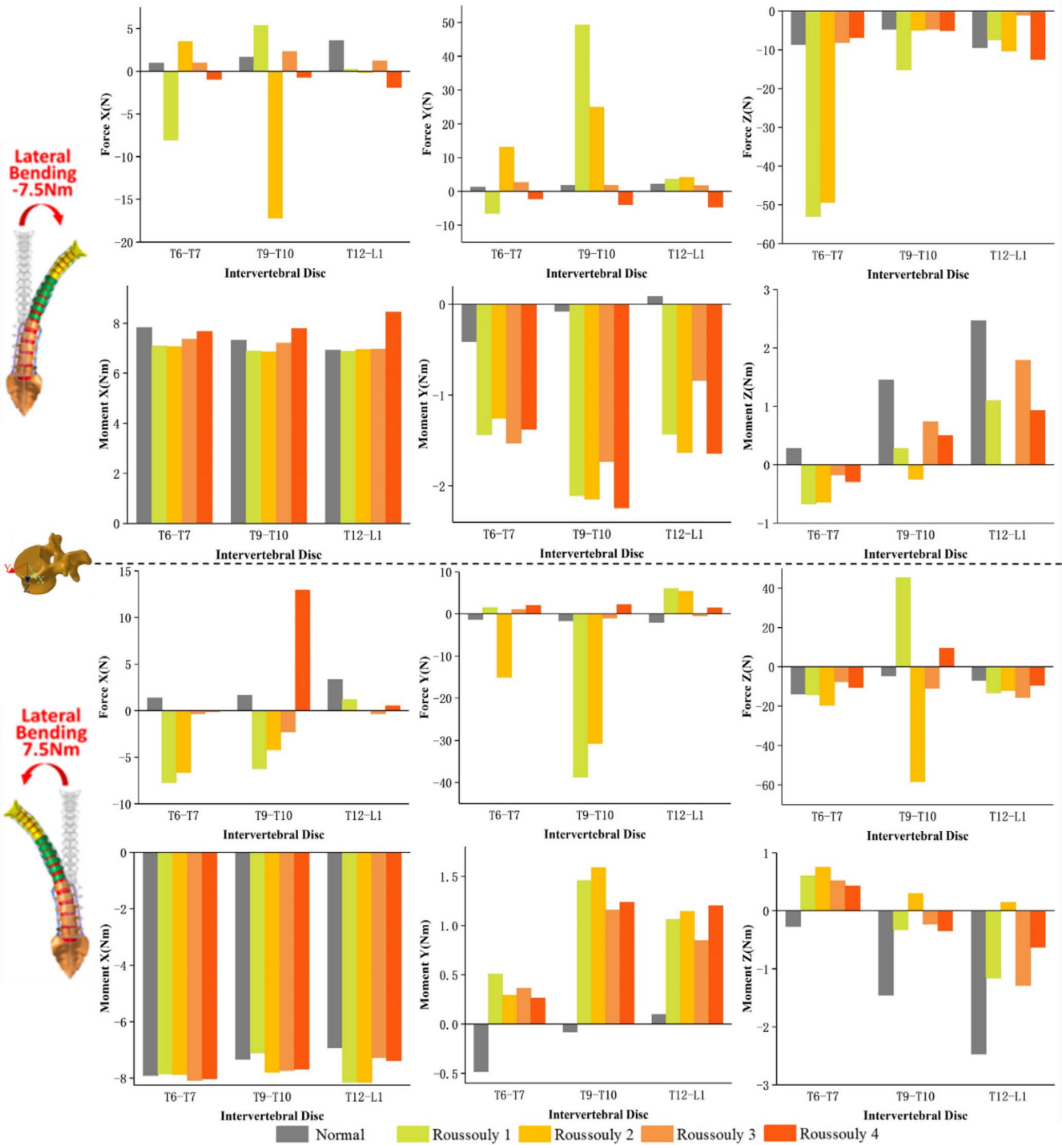
Three‐dimensional forces and moments sustained by the T6–T7, T9–T10, and T12–L1 discs in each model under lateral bending loading.

**FIGURE 7:**
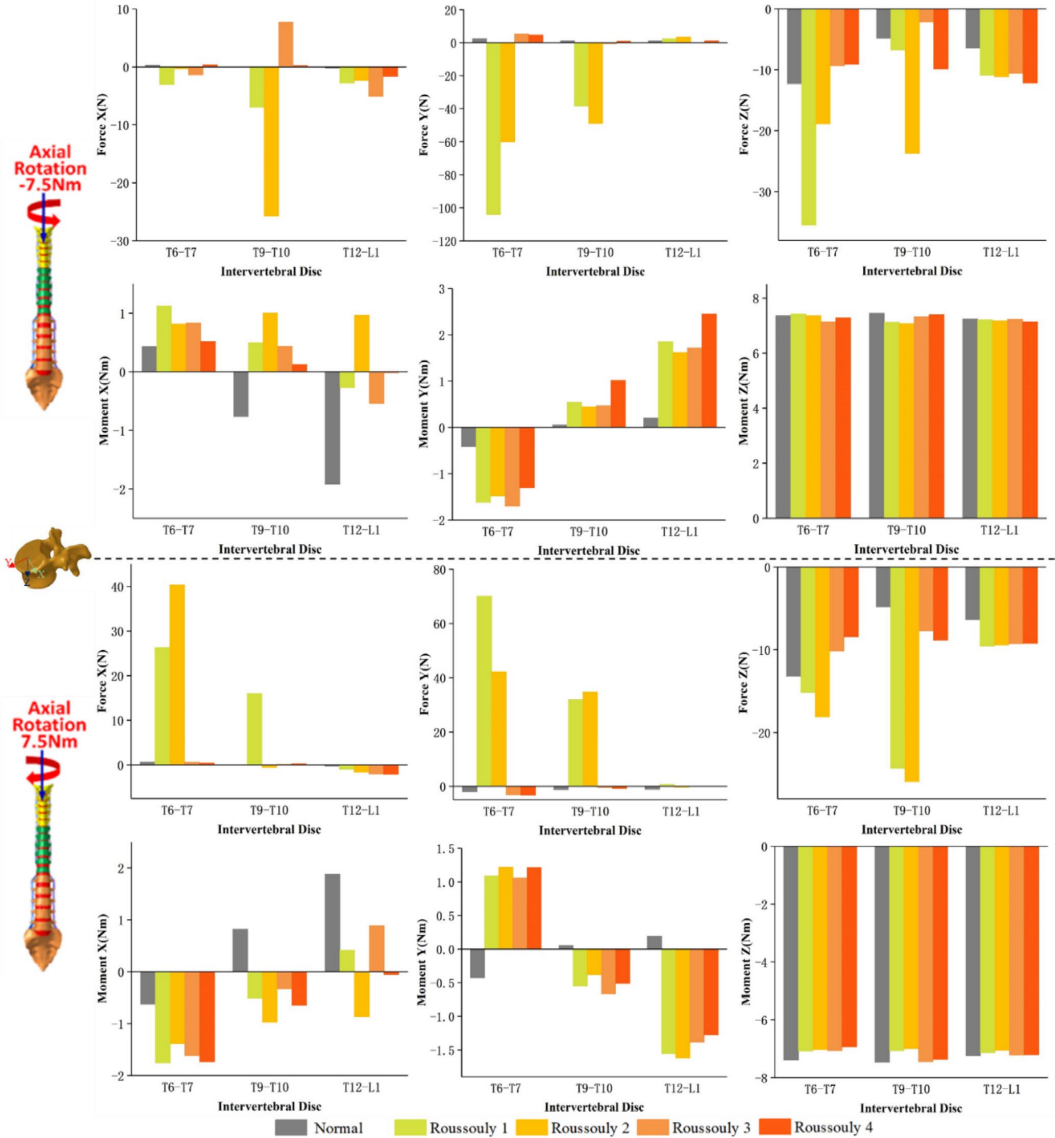
Three‐dimensional forces and moments sustained by the T6–T7, T9–T10, and T12–L1 discs in each model under axial rotation loading.

The force and moment distributions in the coronal, sagittal, and axial planes for the T6–T7, T9–T10, and T12–L1 discs under six loading conditions across different models are presented in Figures 5, 6, 7. Figure 5 illustrates that under 7.5 Nm flexion‐extension, the three‐dimensional forces at the T6–T7 and T9–T10 discs are significantly higher in Roussouly 1 and 2 than in Roussouly 3 and 4. Under flexion‐extension moments, the secondary moments in Normal are smaller than those in the AIS models, particularly along the Z‐axis, where they approach 0 Nm. Compared to the T6–T7 and T12–L1 discs, the secondary moment at the T9–T10 disc in AIS models is the largest along the X‐axis and the smallest along the Z‐axis. In AIS models, the secondary moment around the X‐axis at the T9–T10 disc is highest in Roussouly 1 (−2.2 Nm in flexion and 2.8 Nm in extension) and lowest in Roussouly 3 (−1.8 Nm in flexion and 2.0 Nm in extension). Additionally, Roussouly 2 exhibits the highest secondary moments around the Z‐axis at both the T9–T10 and T12–L1 discs, with 0.6 Nm in flexion and −0.8 Nm in extension at T9–T10, and 1.6 Nm in flexion and −1.3 Nm in extension at T12–L1.

As shown in Figure 6, under 7.5 Nm left bending, Roussouly 1 exhibits greater anterior shear force at the T9–T10 discs (49.3 N) and greater compressive force at the T6–T7 discs (53.0 N) compared to the other models. Additionally, Roussouly 2 exhibits a notably large rightward shear force at the T9–T10 discs (−17.2 N). Under 7.5 Nm right bending, Roussouly 1 and 2 exhibit greater anterior shear and compressive forces at the T9–T10 discs. Compared to the normal model, AIS models show increased secondary moments along the Y‐axis and decreased moments along the Z‐axis at the T9–T10 and T12–L1 discs.

As shown in Figure 7, under 7.5 Nm left rotation, Roussouly 1 exhibits greater shear and compressive forces at the T6–T7 disc compared to the other models. At the T9–T10 disc, Roussouly 2 demonstrates larger shear and compressive forces. Under 7.5 Nm right rotation, the shear force and compressive forces at the T6–T7 and T9–T10 discs are higher in Roussouly 1 and 2 than in Roussouly 3 and 4. Compared to Normal, secondary moments along the Y‐axis are elevated in the AIS models. Additionally, the T9–T10 disc exhibits the smallest secondary moments along the Y‐axis among the three discs across all models.

### 3.3 Intervertebral Disc Stress Analysis

**FIGURE 8:**
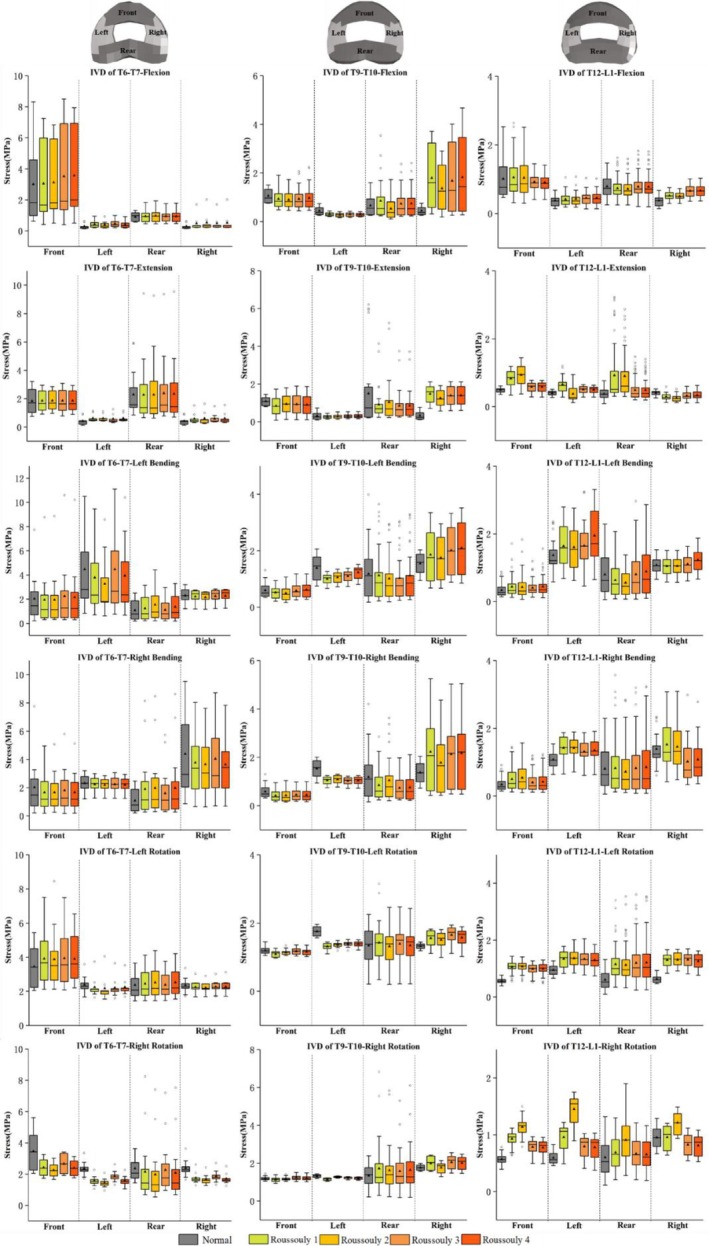
Boxplot of Von Mises stress in the front, rear, left, and right regions of the T6–T7, T9–T10, and T12–L1 intervertebral discs (IVDs) under six loading conditions for each model.

**FIGURE A1:**
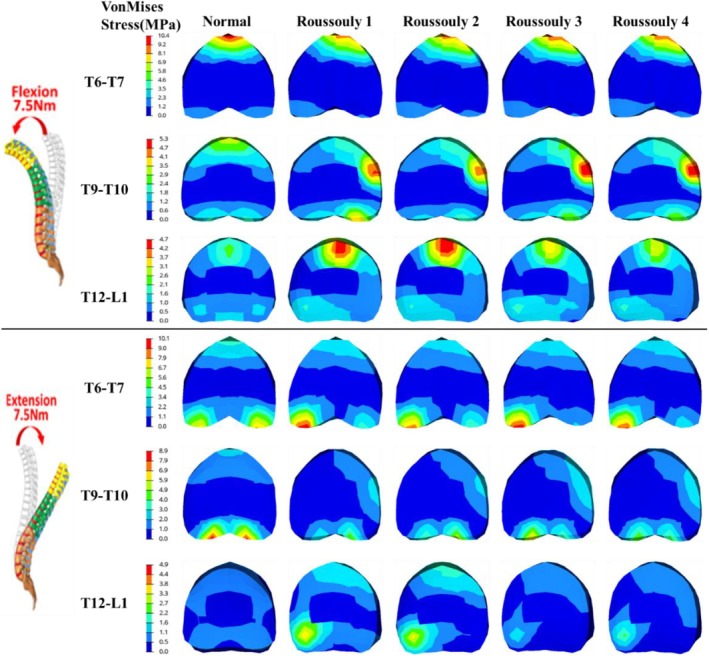
Von Mises stress distribution on the T6–T7, T9–T10, and T12–L1 discs under flexion–extension loading in each model.

**FIGURE A2:**
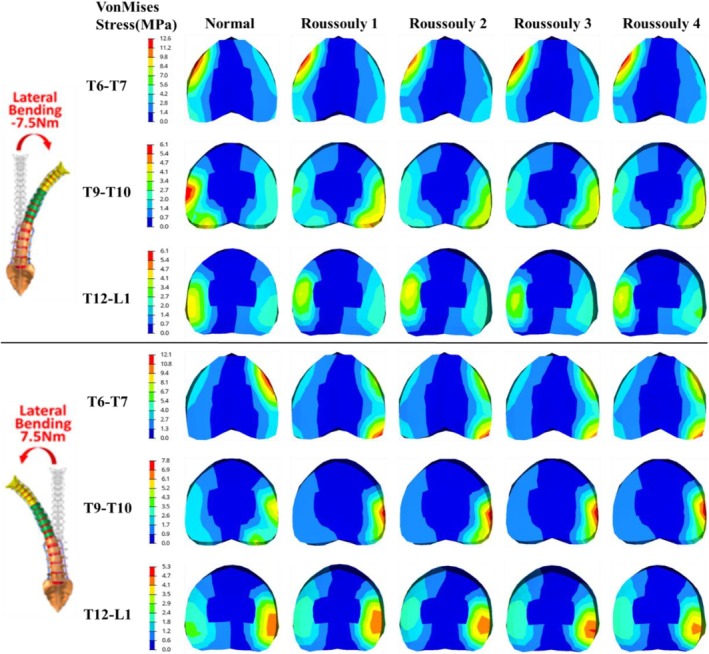
Von Mises stress distribution on the T6–T7, T9–T10, and T12–L1 discs under lateral bending in each model.

**FIGURE A3:**
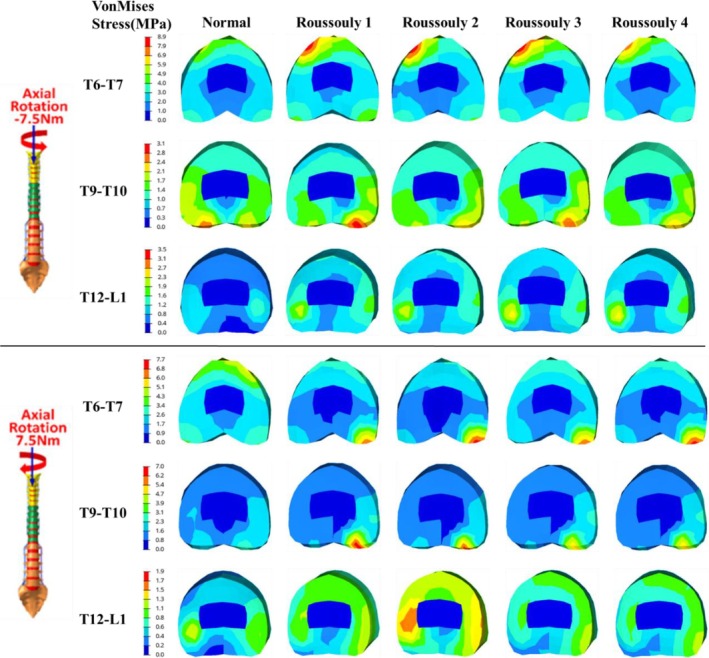
Von Mises stress distribution on the T6–T7, T9–T10, and T12–L1 discs under axial rotation loading in each model.

The Von Mises stress distributions at the T6–T7, T9–T10, and T12–L1 discs under flexion–extension, lateral bending, and axial rotation conditions are shown in Figures A1, A2, A3. Figure 8 shows a more detailed distribution of stress in the front, rear, left, and right regions of each disc. Statistical comparisons of the four disc regions with significant results (*p* < 0.05) among models are provided in Table A2.

As shown in Figures A1 and 8, under the flexion‐extension condition, stress in the T9–T10 disc of the normal model is concentrated in the front and rear, whereas in AIS models, it shifts toward the right. Among these, the stress on the right of the T9–T10 disc in Roussouly 2 is significantly lower than in Roussouly 1, 3, and 4. Under 7.5 Nm flexion, stress on the front of the T6–T7 and T12–L1 discs is the highest in all models. At the front of the T6–T7 disc, Roussouly 4 (3.56 ± 2.69) exhibits significantly higher stress than Roussouly 1 and 2 (3.05 ± 2.44 and 3.12 ± 2.31). For the stress on the front of the T12–L1 disc, Roussouly 3 and 4 (0.92 ± 0.25 and 0.91 ± 0.24) exhibit significantly lower stress than Roussouly 1 and 2 (1.05 ± 0.56 and 1.04 ± 0.55). Under 7.5 Nm extension, stress in the T6–T7 disc is predominantly distributed in the rear across all models. Among these, rear stress in the T6–T7 disc of Roussouly 1 (2.27 ± 2.15) is significantly lower than in Roussouly 3 and 4 (2.39 ± 2.10 and 2.34 ± 2.18). Conversely, rear stress in the T12–L1 disc is significantly higher in Roussouly 1 and 2 (0.92 ± 0.82 and 0.90 ± 0.69) than in Roussouly 3 and 4 (0.49 ± 0.29 and 0.49 ± 0.30).

As shown in Figures A2 and 8, under left and right bending conditions, stress in the T6–T7 and T12–L1 discs of all models is concentrated on the loaded side. During left bending, the stress on the left of the T6–T7 disc in Roussouly 3 (4.47 ± 3.46) is significantly higher than in the other AIS models (3.79 ± 2.83, 3.27 ± 2.48 and 3.94 ± 3.06, respectively). For the T9–T10 disc, the stress in the normal model is primarily concentrated on the left, whereas in AIS models, it shifts toward the right. Among these, the stress on the right of the T9–T10 disc in the AIS model follows the trend: Roussouly 4 > 3 > 1 > 2, with statistically significant differences between each pair of models. Under right bending, for the stress on the right of the T9–T10 disc, Roussouly 2 (1.78 ± 1.28) exhibits significantly lower stress than the other three models (2.23 ± 1.68, 2.13 ± 1.45, and 2.18 ± 1.52, respectively). On the right of the T12–L1 disc, stress in Roussouly 1 and 2 (1.52 ± 0.73 and 1.46 ± 0.66) is significantly higher than in Roussouly 3 and 4 (1.02 ± 0.63 and 1.08 ± 0.61).

During left rotation, stress in the T6–T7, T9–T10, and T12–L1 discs for all models is concentrated at the front, rear, and left, respectively (Figures A3 and 8). Among these, for the left stress in the T12–L1 disc, Roussouly 4 (1.30 ± 0.27) is significantly lower than the other AIS models (1.34 ± 0.29, 1.36 ± 0.32 and 1.33 ± 0.30, respectively). Under right rotation, the stress in the T6–T7 disc in Normal is biased toward the front, while the AIS models tend to shift toward the rear, with Roussouly 2 (1.97 ± 1.74) exhibiting significantly lower stress than the other AIS models (2.15 ± 1.90, 2.27 ± 1.57 and 2.08 ± 1.73, respectively). In the T9–T10 disc, stress is concentrated in the rear across all models. For the T12–L1 disc, comparisons within the AIS models show that the stress follows the trend: Roussouly 2 > 1 > 3 > 4.

## 4 Discussion

This study utilized FE simulation to systematically evaluate the biomechanical response of AIS models with different Roussouly types in terms of spinal ROM and intervertebral disc loadings. To ensure comparability and minimize confounding factors, mesh morphing was applied to an experimentally validated normal model to construct AIS models representing Roussouly types 1–4, thereby enhancing the accuracy and reliability of the findings. The results highlight the significant biomechanical role of sagittal alignment in AIS and suggest that the Roussouly classification, beyond the widely studied coronal deformities, warrants greater clinical consideration. Furthermore, significant biomechanical differences were observed between Roussouly types 1 and 2, compared to types 3 and 4.

This study revealed significant differences in movement patterns between the Lenke 1 AIS model and the normal model, based on changes in segmental and overall spinal ROM. The results indicate significant left–right asymmetry in the ROM of the main thoracic curve (T7–T12) in the AIS model, with right‐bending ROM only 68.1%–70.9% of left bending, suggesting that scoliosis restricts rightward bending in this region. However, the overall (T1–S1) ROM is more symmetrical, with the left‐to‐right bending ROM ratio increasing to 82.7%–96.1%, likely due to compensatory effects from the T1–T6 and L1–S1 segments [15]. These adaptations aim to preserve global spinal balance by enhancing mobility in less‐affected regions, particularly when sagittal imbalance causes the lumbar spine to adjust its curvature. Such compensations offset the asymmetric loading and motion resulting from thoracic structural deformities and vertebral rotation in AIS [25]. Significant differences in ROM are also observed among the different Roussouly types of AIS models. As shown in Figure 2, Roussouly 2 exhibits lower ROM in the main thoracic segment, with only 9.6° during right bending. This may be due to its smaller thoracic curve and straighter spinal morphology, which could limit movement in this region [8]. Due to the smaller lumbar lordosis in Roussouly type 2, compensatory motion tends to occur more in the upper thoracic region (T1–T6) to maintain mechanical balance, as shown in Figure 3. This suggests that clinical interventions should focus on restoring mobility in the scoliotic segments and minimizing excessive motion in the upper thoracic region. Similarly, Roussouly 1 shows increased coupling motion in the T1–T12 segment, which may also be related to its smaller lumbar lordosis, as shown in Figure 3 [8]. Roussouly type 1 has a smaller lumbar lordosis, and the thoracic spine may compensate with kyphosis to maintain sagittal balance, thereby increasing the risk of proximal junctional kyphosis [26]. Moreover, AIS patients with Roussouly type 1 have the highest risk of poor sagittal shape recovery following thoracic curve correction surgery [27]. Therefore, during corrective surgery or rehabilitation for AIS patients with Roussouly type 1, particular attention should be paid to the biomechanical characteristics of the thoracic vertebrae to reduce the incidence of proximal junctional kyphosis. In terms of overall ROM and primary motions of the L1–S1 segment, Roussouly 3 and 4 exhibit lower mobility compared to Roussouly 1 and 2, suggesting a more controlled motion pattern that may help mitigate excessive disc loadings and support better mechanical balance. Lumbar curvature was identified as a key determinant of the Roussouly classification by Wang et al. [28]. They also constructed FE models of the normal lumbar spine for Roussouly types 1–4 using mesh morphing. The results showed that the straighter Roussouly types 1 and 2 exhibited increased spinal mobility, consistent with these findings. Therefore, optimizing motion regulation should be a key focus in the treatment of AIS patients with Roussouly types 1 and 2, in order to reduce the impact of mechanical imbalance on disease progression and outcomes.

The asymmetry in the ROM of the AIS spine not only affects overall movement patterns but also leads to significant changes in secondary moments, further exacerbating the mechanical imbalance in the spine, as shown in Figures 5, 6, 7. The abnormal increase in local moments may be related to the rotational deformity of the AIS spine and the increased scoliosis curvature, resulting in local uneven loading. The results of this study indicate that secondary moments in the AIS model are significantly higher than those in the normal model, in contrast to the negligible secondary moments observed in the normal model under flexion and extension conditions. The increase in abnormal moments is primarily concentrated in the T9–T10 and T12–L1 discs during right bending, suggesting that the adjacent discs near the apex and lower‐end vertebrae may experience greater biomechanical loadings and could be key areas for disease progression. Comparative analysis among the AIS models further reveals differences in mechanical loads across various Roussouly types. The study found that Roussouly 1 and 2 exhibit significantly higher shear and compressive forces at the T6–T7 and T9–T10 discs compared to Roussouly 3 and 4, consistent with the overall spinal ROM trends. This is likely due to the smaller lumbar lordosis and weaker sagittal balance in Roussouly types 1 and 2, which cause the thoracic discs to require a greater local load to maintain stability [8, 26]. Specifically, the discs near the apex show an increase in shear force and a decrease in compressive force, likely related to the complex coupling compensation mechanisms of vertebral rotation and coronal plane scoliosis at the apex region. Further analysis reveals differences in the loading patterns at the T9–T10 disc between Roussouly 1 and 2: Roussouly 1 exhibits greater shear force, while Roussouly 2 primarily experiences greater compressive force. This difference may be attributed to the larger thoracic kyphosis in Roussouly type 1, which causes the thoracic spine to experience higher shear stress while maintaining sagittal balance. In contrast, Roussouly type 2, with its flatback feature, results in greater compressive loading in this region [8, 26]. Therefore, treatment strategies for patients with Roussouly types 1 and 2 should fully consider their shear and compressive forces at the T6–T7 and T9–T10 discs to improve therapeutic outcomes and minimize the risk of disease progression.

This study conducted a detailed analysis of the disc Von Mises stress distribution and found that under different loading conditions, stress in the normal model tends to concentrate on the loaded side. In contrast, in the AIS model, stress in the T9–T10 disc is more concentrated on the right (concave) side, which is consistent with the higher mechanical load borne by the concave side growth plate in AIS patients [29]. A study developed patient‐specific FE models based on 5 AIS patients (4 with Lenke type 1 and 1 with Lenke type 2) and found, in a three‐year follow‐up, that disc stress on the concave side of the scoliosis was consistently higher than that on the convex side [30]. Similarly, another study examined the biomechanical changes in AIS lumbar segments under different loading conditions using patient‐specific FE models and observed that stress was primarily concentrated on the concave side [18]. Prolonged high stress on the concave side may result from the unique structural abnormalities of the AIS spine, leading to uneven load distribution and exacerbating the local mechanical load imbalance on the discs. It is worth noting that sustained high stress may induce a series of biological responses, including increased cell apoptosis, upregulation of inflammatory factors, and degradation of proteoglycans, which can affect the mechanical properties of the discs and potentially accelerate the degeneration process [31]. Further analysis of the impact of different Roussouly types on Von Mises stress distribution revealed that the Von Mises stress in the T6–T7 and T9–T10 discs is relatively higher in Roussouly 3 and 4, while stress in the T12–L1 discs is more significant in Roussouly 1 and 2. The T12–L1 discs, as the transition area between the thoracic and lumbar, have loading characteristics that reflect, to some extent, the overall mechanical trend of the lumbar spine. In a study of 331 patients with low back pain and degenerative spinal sagittal morphology, it was found that stress in Roussouly types 1 and 2 primarily concentrates in the disc region, making them more prone to disc herniation and multi‐segment degeneration [32]. In contrast, stress in Roussouly types 3 and 4 is primarily distributed in the posterior spinal structures, increasing the risk of lumbar spondylolisthesis and pars defects. The sagittal morphology of Roussouly types 1 and 2 is relatively flat, with smaller lumbar lordosis, leading to higher axial loads on the lower lumbar segments, thus increasing the risk of lumbar disc herniation. Accordingly, treatment strategies for AIS patients with different Roussouly types should selectively emphasize protecting intervertebral discs or posterior column structures to achieve effective load modulation and prevent degeneration.

In clinical interventions, special attention should be paid to spinal mobility and intervertebral disc loadings in patients with Roussouly types 1 and 2 AIS. Due to smaller lumbar lordosis, these patients are more likely to experience excessive shear forces in the thoracic spine and concentrated compressive forces in the lumbar spine, increasing the risk of proximal junctional kyphosis and lumbar disc degeneration. Treatment strategies should focus on regulating the transfer of shear and compressive forces in the thoracic region. In contrast, patients with Roussouly types 3 and 4 exhibit more controlled spinal motion overall. Due to the low incidence of thoracic disc degeneration, the need for protection of the thoracic discs is relatively low, although the potential risk of damage to lumbar posterior column structures should still be considered.

Although the results of this study offer valuable insights into the biomechanical characteristics of AIS, some limitations still exist. First, the AIS FE model constructed in this study is idealized. Although adjustments have been made to the coronal, sagittal, and vertebral rotation angles based on existing literature to enhance the model's representation of real pathological features, individual anatomical variations have not been fully considered. Therefore, future research could integrate musculoskeletal dynamics modeling, incorporating patient‐specific data (such as individualized imaging parameters and kinematic data) into the FE model to further enhance the model's clinical applicability. Second, regarding the selection of the control model, given that previous studies have systematically examined biomechanical differences between AIS patients and normal individuals [19, 28], and since this study focuses on analyzing the biomechanical characteristics of AIS patients with different Roussouly types, only a normal model of Roussouly type 3 was constructed as a control. This representative model was considered sufficient to serve as a baseline reference for evaluating the biomechanical variations across AIS subtypes. Moreover, although Von Mises stress is not theoretically the optimal parameter for anisotropic materials, it is widely accepted and used in spinal FE simulations—particularly for intervertebral discs—due to its ability to integrate multi‐axial loading components into a single scalar value [33, 34]. This makes it effective in capturing overall stress exposure and identifying regions susceptible to mechanical failure or degeneration, thereby providing a practical and interpretable metric for assessing stress concentration trends. Finally, this study was based solely on Lenke type 1 for analysis, but differences may still exist among different Lenke types in terms of coronal curvature, rotational deformity, and sagittal compensation mechanisms. Therefore, future studies should include additional Lenke types to more comprehensively understand the mechanical adaptation mechanisms of AIS and provide more detailed biomechanical evidence for personalized diagnosis and treatment.

## 5 Conclusions

This study systematically evaluated the biomechanical characteristics of spinal ROM and intervertebral disc loadings in Lenke type 1 AIS models with different Roussouly classifications, highlighting the key role of sagittal morphology in spinal mechanical adaptation. The study further clarified the specific differences between Roussouly types in motion patterns and load distribution, offering valuable biomechanical evidence for personalized diagnosis and treatment of AIS. The results demonstrate that the AIS spinal motion pattern exhibits significant asymmetry compared to the normal model, particularly in the main thoracic curve segment. This is accompanied by an abnormal concentration of intervertebral disc loadings and a significant increase in secondary moments, including elevated shear and compressive forces. Further analysis of different Roussouly types reveals that, compared to Roussouly 3 and 4, Roussouly 1 and 2 exhibit increased spinal mobility, with significantly higher shear and compressive forces on the intervertebral discs adjacent to the apex and upper end vertebrae. Significant differences in intervertebral disc stress were also observed across Roussouly types. Von Mises stress on the intervertebral discs adjacent to the apex and upper end vertebrae is higher in Roussouly 3 and 4, whereas stress on the intervertebral discs adjacent to the lower vertebrae is more pronounced in Roussouly 1 and 2. Clinically, this suggests that Roussouly types 1 and 2 may be at greater risk for disc degeneration and decreased mechanical stability at the lower lumbar levels, while types 3 and 4 may require greater attention at the upper levels of the spine. Therefore, during corrective treatment, rehabilitation training, and brace therapy of AIS, special attention should be given to the biomechanical characteristic differences across Roussouly classifications. Personalized intervention strategies should be optimized to enhance spinal mechanical stability, reduce mechanical imbalance, and effectively manage thoracic shear and compressive forces, ultimately improving outcomes.

## Author Contributions

Zhihua Wu conceptualized the study, conducted data curation, formal analysis, investigation, methodology development, validation, visualization, and wrote the original draft. Huantong Cheng, Jia He, Xiaowei Dai, De Liang, Xiaobing Jiang, Yueli Sun, Ruitao She, and Yuanfang Lin contributed to data curation, formal analysis, methodology, validation, and manuscript review and editing. Junyu He and Ziyang Liang contributed to formal analysis, methodology, validation, and manuscript review and editing. Ziyang Liang also acquired funding. Wei Wei contributed to data curation, formal analysis, investigation, methodology, validation, and manuscript review and editing. All authors reviewed and approved the final manuscript.

## Conflicts of Interest

The authors declare no conflicts of interest.

## Data Availability

The data that support the findings of this study are available from the corresponding author upon reasonable request.

## Appendix A

**TABLE A1:** Spinal parameters across the three anatomical planes for each finite element model.

Angle (°)	Normal	Roussouly 1	Roussouly 2	Roussouly 3	Roussouly 4
Coronal plane					
Cobb (T7–T12)	0	28	28	28	28
L1–L4	0	13	13	13	13
Sagittal plane					
T1 slope	27	27	27	27	27
UTK (T2–T5)	18	18	18	18	18
TK (T5–T12)	36	34	25	36	34
TLK (T10–L2)	9	4	4	9	9
LL (L1–S1)	49	38	36	49	54
SS	39	28	33	39	49
Axial plane					
T1	0	4	4	4	4
T2	0	4.8	4.8	4.8	4.8
T3	0	7.5	7.5	7.5	7.5
T4	0	7.5	7.5	7.5	7.5
T5	0	8	8	8	8
T6	0	8.2	8.2	8.2	8.2
T7	0	9.9	9.9	9.9	9.9
T8	0	12.9	12.9	12.9	12.9
T9	0	13.8	13.8	13.8	13.8
T10	0	12.6	12.6	12.6	12.6
T11	0	12.3	12.3	12.3	12.3
T12	0	11	11	11	11
L1	0	10.9	10.9	10.9	10.9
L2	0	10	10	10	10
L3	0	9.3	9.3	9.3	9.3
L4	0	7.8	7.8	7.8	7.8
L5	0	5.7	5.7	5.7	5.7
S1	0	3.6	3.6	3.6	3.6

Abbreviations: LL, lumbar lordosis; SS, sacral slope; TK, thoracic kyphosis; TLK, thoracolumbar kyphosis; UTK, upper thoracic kyphosis.

**TABLE A2:** Statistical comparisons of the four disc regions among models with significant differences.

Region	Versus	T	P
T6–T7‐Flexion‐Front	Roussouly 1 vs. 3	−3.1405	0.0094
	Roussouly 1 vs. 4	−5.8258	0.0001
	Roussouly 2 vs. 4	−3.5143	0.0048
T6–T7‐Flexion‐Left	Normal vs. Roussouly 3	−2.3651	0.0456
	Roussouly 1 vs. 2	2.4353	0.0409
	Roussouly 1 vs. 3	−3.9904	0.0040
	Roussouly 1 vs. 4	3.4002	0.0094
	Roussouly 2 vs. 3	−3.1891	0.0128
	Roussouly 3 vs. 4	3.8207	0.0051
T6–T7‐Flexion‐Rear	Roussouly 1 vs. 2	−2.9249	0.0095
	Roussouly 2 vs. 4	2.7384	0.0140
T9–T10‐Flexion‐Front	Roussouly 1 vs. 2	2.3582	0.0272
	Roussouly 3 vs. 4	−2.8947	0.0082
T9–T10‐Flexion‐Left	Normal vs. Roussouly 3	1.8198	0.0005
	Roussouly 1 vs. 2	6.4984	0.0000
	Roussouly 1 vs. 4	13.8109	0.0000
	Roussouly 2 vs. 3	−2.3498	0.0385
	Roussouly 2 vs. 4	−2.7003	0.0206
T9–T10‐Flexion‐Rear	Roussouly 1 vs. 2	4.4684	0.0001
	Roussouly 1 vs. 3	2.6681	0.0120
	Roussouly 1 vs. 4	2.6138	0.0137
	Roussouly 2 vs. 3	−5.9667	0.0000
	Roussouly 2 vs. 4	−5.6296	0.0000
T9–T10‐Flexion‐Right	Normal vs. Roussouly 3	−3.3427	0.0066
	Roussouly 1 vs. 2	3.8588	0.0027
	Roussouly 2 vs. 3	−2.2215	0.0482
	Roussouly 2 vs. 4	−2.5025	0.0294
	Roussouly 3 vs. 4	−2.4200	0.0340
T12–L1‐Flexion‐Front	Roussouly 1 vs. 3	2.5742	0.0130
	Roussouly 1 vs. 4	2.8013	0.0072
	Roussouly 2 vs. 3	2.4627	0.0172
	Roussouly 2 vs. 4	2.6998	0.0094
	Roussouly 3 vs. 4	6.1033	0.0000
T12–L1‐Flexion‐Left	Normal vs. Roussouly 3	−2.2582	0.0347
T12–L1‐Flexion‐Rear	Roussouly 1 vs. 2	4.9458	0.0000
	Roussouly 1 vs. 3	−4.2296	0.0001
	Roussouly 1 vs. 4	−4.0273	0.0002
	Roussouly 2 vs. 3	−4.6330	0.0000
	Roussouly 2 vs. 4	−4.4798	0.0000
T12–L1‐Flexion‐Right	Normal vs. Roussouly 3	−8.6744	0.0000
	Roussouly 1 vs. 2	3.8712	0.0009
	Roussouly 1 vs. 3	−10.9172	0.0000
	Roussouly 1 vs. 4	−11.8872	0.0000
	Roussouly 2 vs. 3	−9.6552	0.0000
	Roussouly 2 vs. 4	−10.4797	0.0000
	Roussouly 3 vs. 4	−4.8721	0.0001
T6–T7‐Extension‐Left	Normal vs. Roussouly 3	−4.2269	0.0029
T6–T7‐Extension‐Rear	Roussouly 1 vs. 3	−2.5998	0.0187
	Roussouly 1 vs. 4	−4.4650	0.0003
T6–T7‐Extension‐Right	Roussouly 1 vs. 4	−3.3684	0.0098
T9–T10‐Extension‐Front	Roussouly 1 vs. 2	−13.5726	0.0000
	Roussouly 1 vs. 3	−8.6838	0.0000
	Roussouly 1 vs. 4	−7.1479	0.0000
	Roussouly 2 vs. 4	4.2441	0.0003
	Roussouly 3 vs. 4	11.6888	0.0000
T9–T10‐Extension‐Left	Roussouly 1 vs. 2	−2.3325	0.0397
	Roussouly 1 vs. 3	−8.1986	0.0000
	Roussouly 1 vs. 4	−8.0478	0.0000
	Roussouly 2 vs. 3	−11.9961	0.0000
	Roussouly 2 vs. 4	−13.9845	0.0000
	Roussouly 3 vs. 4	−3.0710	0.0106
T9–T10‐Extension‐Rear	Normal vs. Roussouly 3	3.1236	0.0039
	Roussouly 2 vs. 3	2.7642	0.0095
	Roussouly 2 vs. 4	2.7010	0.0111
T9–T10‐Extension‐Right	Normal vs. Roussouly 3	−7.9090	0.0000
	Roussouly 1 vs. 2	4.3652	0.0011
	Roussouly 2 vs. 3	−4.8408	0.0005
	Roussouly 2 vs. 4	−5.1400	0.0003
T12–L1‐Extension‐Front	Normal vs. Roussouly 3	−6.6227	0.0000
	Roussouly 1 vs. 2	−7.7492	0.0000
	Roussouly 1 vs. 3	15.1132	0.0000
	Roussouly 1 vs. 4	14.8129	0.0000
	Roussouly 2 vs. 3	12.6603	0.0000
	Roussouly 2 vs. 4	12.6690	0.0000
T12–L1‐Extension‐Left	Normal vs. Roussouly 3	−4.5581	0.0002
	Roussouly 1 vs. 2	9.9410	0.0000
	Roussouly 1 vs. 3	3.7121	0.0013
	Roussouly 1 vs. 4	4.1193	0.0005
	Roussouly 2 vs. 3	−3.0056	0.0067
	Roussouly 2 vs. 4	−2.9544	0.0076
	Roussouly 3 vs. 4	2.6193	0.0160
T12–L1‐Extension‐Rear	Normal vs. Roussouly 3	−4.0892	0.0001
	Roussouly 1 vs. 3	6.1522	0.0000
	Roussouly 1 vs. 4	6.3819	0.0000
	Roussouly 2 vs. 3	7.7036	0.0000
	Roussouly 2 vs. 4	7.9193	0.0000
T12–L1‐Extension‐Right	Normal vs. Roussouly 3	3.1735	0.0046
	Roussouly 1 vs. 2	2.5204	0.0199
	Roussouly 1 vs. 4	−2.8523	0.0095
	Roussouly 2 vs. 3	−2.4721	0.0221
	Roussouly 2 vs. 4	−2.9927	0.0069
	Roussouly 3 vs. 4	−7.6824	0.0000
T6–T7‐Left Bending‐Front	Roussouly 3 vs. 4	2.5306	0.0279
T6–T7‐Left Bending‐Left	Roussouly 1 vs. 2	3.2493	0.0117
	Roussouly 1 vs. 3	−2.9029	0.0198
	Roussouly 2 vs. 3	−3.1469	0.0137
	Roussouly 2 vs. 4	−3.0369	0.0131
	Roussouly 3 vs. 4	2.8373	0.0219
T6–T7‐Left Bending‐Rear	Roussouly 1 vs. 2	−2.5957	0.0188
	Roussouly 1 vs. 3	2.3533	0.0309
	Roussouly 1 vs. 4	−2.7596	0.0134
	Roussouly 2 vs. 3	2.5460	0.0209
	Roussouly 2 vs. 4	2.4372	0.0261
	Roussouly 3 vs. 4	−2.5935	0.0189
T6–T7‐Left Bending‐Right	Roussouly 1 vs. 2	3.0830	0.0150
	Roussouly 1 vs. 3	−3.4358	0.0089
	Roussouly 1 vs. 4	−17.3925	0.0000
	Roussouly 2 vs. 3	−3.2654	0.0114
	Roussouly 2 vs. 4	−6.8154	0.0001
T9–T10‐Left Bending‐Front	Roussouly 1 vs. 3	−3.9662	0.0006
	Roussouly 1 vs. 4	−7.5409	0.0000
	Roussouly 2 vs. 3	−11.5457	0.0000
	Roussouly 2 vs. 4	−17.9749	0.0000
	Roussouly 3 vs. 4	−3.3154	0.0030
T9–T10‐Left Bending‐Left	Normal vs. Roussouly 3	3.8084	0.0029
	Roussouly 1 vs. 2	−3.7298	0.0033
	Roussouly 1 vs. 3	−11.2538	0.0000
	Roussouly 1 vs. 4	−12.5141	0.0000
	Roussouly 2 vs. 3	−2.6940	0.0209
	Roussouly 2 vs. 4	−7.2361	0.0000
	Roussouly 3 vs. 4	−11.4398	0.0000
T9–T10‐Left Bending‐Rear	Roussouly 1 vs. 2	2.1524	0.0393
	Roussouly 1 vs. 3	2.1390	0.0404
	Roussouly 2 vs. 4	−4.2961	0.0002
	Roussouly 3 vs. 4	−6.5093	0.0000
T9–T10‐Left Bending‐Right	Normal vs. Roussouly 3	−2.9568	0.0130
	Roussouly 1 vs. 2	2.2421	0.0465
	Roussouly 1 vs. 3	−4.5896	0.0008
	Roussouly 1 vs. 4	−9.8844	0.0000
	Roussouly 2 vs. 3	−9.2214	0.0000
	Roussouly 2 vs. 4	−7.6934	0.0000
	Roussouly 3 vs. 4	−3.7919	0.0030
T12–L1‐Left Bending‐Front	Normal vs. Roussouly 3	−6.1368	0.0000
	Roussouly 3 vs. 4	−3.1463	0.0028
T12–L1‐Left Bending‐Left	Normal vs. Roussouly 3	−3.0468	0.0061
	Roussouly 1 vs. 4	−4.7307	0.0001
	Roussouly 2 vs. 4	−3.4841	0.0022
	Roussouly 3 vs. 4	−3.2423	0.0039
T12–L1‐Left Bending‐Rear	Roussouly 1 vs. 2	4.5276	0.0000
	Roussouly 1 vs. 3	−4.4278	0.0000
	Roussouly 1 vs. 4	−6.4359	0.0000
	Roussouly 2 vs. 3	−4.7692	0.0000
	Roussouly 2 vs. 4	−6.1698	0.0000
	Roussouly 3 vs. 4	−4.5952	0.0000
T12–L1‐Left Bending‐Right	Roussouly 1 vs. 3	−4.8976	0.0001
	Roussouly 1 vs. 4	−9.0221	0.0000
	Roussouly 2 vs. 3	−3.6533	0.0015
	Roussouly 2 vs. 4	−7.7458	0.0000
	Roussouly 3 vs. 4	−12.1500	0.0000
T6–T7‐Right Bending‐Front	Roussouly 2 vs. 3	−2.2958	0.0423
	Roussouly 2 vs. 4	2.5824	0.0255
T6–T7‐Right Bending‐Left	Normal vs. Roussouly 3	3.7469	0.0056
	Roussouly 1 vs. 2	3.0936	0.0148
	Roussouly 1 vs. 4	6.4000	0.0002
T6–T7‐Right Bending‐Rear	Normal vs. Roussouly 3	−2.2377	0.0389
	Roussouly 1 vs. 2	−2.5054	0.0227
	Roussouly 1 vs. 4	−2.2929	0.0349
	Roussouly 2 vs. 3	2.1981	0.0421
T9–T10‐Right Bending‐Front	Normal vs. Roussouly 3	4.0667	0.0005
	Roussouly 1 vs. 3	−10.8304	0.0000
	Roussouly 1 vs. 4	−6.9751	0.0000
	Roussouly 2 vs. 3	−2.8732	0.0086
	Roussouly 2 vs. 4	−2.5168	0.0193
	Roussouly 3 vs. 4	3.0850	0.0052
T9–T10‐Right Bending‐Left	Normal vs. Roussouly 3	8.5487	0.0000
	Roussouly 1 vs. 2	−10.5760	0.0000
	Roussouly 1 vs. 3	3.0858	0.0104
	Roussouly 2 vs. 3	8.8553	0.0000
	Roussouly 2 vs. 4	8.4071	0.0000
	Roussouly 3 vs. 4	−6.1308	0.0001
T9–T10‐Right Bending‐Rear	Normal vs. Roussouly 3	3.0954	0.0041
	Roussouly 1 vs. 2	−2.1043	0.0436
	Roussouly 1 vs. 3	3.2450	0.0028
	Roussouly 1 vs. 4	3.1536	0.0036
	Roussouly 2 vs. 3	2.9025	0.0068
	Roussouly 2 vs. 4	2.8379	0.0079
T9–T10‐Right Bending‐Right	Normal vs. Roussouly 3	−2.2996	0.0421
	Roussouly 1 vs. 2	3.4119	0.0058
	Roussouly 2 vs. 3	−2.5968	0.0248
	Roussouly 2 vs. 4	−4.6171	0.0007
T12–L1‐Right Bending‐Front	Normal vs. Roussouly 3	−2.6860	0.0097
	Roussouly 1 vs. 2	−4.2938	0.0001
	Roussouly 1 vs. 3	7.6183	0.0000
	Roussouly 1 vs. 4	7.9127	0.0000
	Roussouly 2 vs. 3	6.7569	0.0000
	Roussouly 2 vs. 4	7.0244	0.0000
	Roussouly 3 vs. 4	−4.8671	0.0000
T12–L1‐Right Bending‐Left	Normal vs. Roussouly 3	−4.1784	0.0004
	Roussouly 1 vs. 3	5.4189	0.0000
	Roussouly 1 vs. 4	4.0950	0.0005
	Roussouly 2 vs. 3	3.4423	0.0024
	Roussouly 2 vs. 4	2.4409	0.0236
	Roussouly 3 vs. 4	−13.9596	0.0000
T12–L1‐Right Bending‐Rear	Roussouly 1 vs. 2	4.6481	0.0000
	Roussouly 2 vs. 3	−4.2921	0.0001
	Roussouly 2 vs. 4	−4.9066	0.0000
	Roussouly 3 vs. 4	−4.3634	0.0000
T12–L1‐Right Bending‐Right	Normal vs. Roussouly 3	3.1889	0.0044
	Roussouly 1 vs. 3	5.3674	0.0000
	Roussouly 1 vs. 4	5.3044	0.0000
	Roussouly 2 vs. 3	6.2458	0.0000
	Roussouly 2 vs. 4	6.0982	0.0000
	Roussouly 3 vs. 4	−3.1134	0.0053
T6–T7‐Left Rotation‐Front	Normal vs. Roussouly 3	−2.3917	0.0358
T6–T7‐Left Rotation‐Left	Normal vs. Roussouly 3	3.8447	0.0049
	Roussouly 1 vs. 3	2.7390	0.0255
T6–T7‐Left Rotation‐Rear	Roussouly 1 vs. 2	−2.2379	0.0389
	Roussouly 1 vs. 3	2.3540	0.0309
	Roussouly 1 vs. 4	−8.4827	0.0000
	Roussouly 2 vs. 3	2.6176	0.0180
	Roussouly 3 vs. 4	−5.5241	0.0000
T6–T7‐Left Rotation‐Right	Roussouly 1 vs. 2	2.4735	0.0385
	Roussouly 2 vs. 3	−3.3771	0.0087
	Roussouly 2 vs. 4	−2.5855	0.0323
T9–T10‐Left Rotation‐Front	Roussouly 1 vs. 2	−4.1956	0.0003
	Roussouly 1 vs. 3	−12.4916	0.0000
	Roussouly 1 vs. 4	−8.8495	0.0000
	Roussouly 2 vs. 3	−3.3916	0.0025
	Roussouly 3 vs. 4	4.1430	0.0004
T9–T10‐Left Rotation‐Left	Normal vs. Roussouly 3	11.7226	0.0000
	Roussouly 1 vs. 2	−4.4159	0.0010
	Roussouly 1 vs. 3	−9.4993	0.0000
	Roussouly 1 vs. 4	−9.1811	0.0000
	Roussouly 2 vs. 3	−5.0455	0.0004
	Roussouly 2 vs. 4	−2.5236	0.0283
T9–T10‐Left Rotation‐Rear	Roussouly 1 vs. 2	3.1425	0.0037
	Roussouly 1 vs. 4	2.8506	0.0077
	Roussouly 2 vs. 3	−5.2898	0.0000
	Roussouly 3 vs. 4	11.0421	0.0000
T9–T10‐Left Rotation‐Right	Normal vs. Roussouly 3	−5.8741	0.0001
	Roussouly 1 vs. 3	−5.7232	0.0001
	Roussouly 2 vs. 3	−4.7883	0.0006
	Roussouly 2 vs. 4	−3.1902	0.0086
	Roussouly 3 vs. 4	6.6270	0.0000
T12–L1‐Left Rotation‐Front	Normal vs. Roussouly 3	−16.2709	0.0000
	Roussouly 1 vs. 3	7.3650	0.0000
	Roussouly 1 vs. 4	7.5194	0.0000
	Roussouly 2 vs. 3	7.0702	0.0000
	Roussouly 2 vs. 4	5.8704	0.0000
T12–L1‐Left Rotation‐Left	Normal vs. Roussouly 3	−5.2481	0.0000
	Roussouly 1 vs. 4	2.7620	0.0117
	Roussouly 2 vs. 4	3.0749	0.0057
	Roussouly 3 vs. 4	3.0239	0.0065
T12–L1‐Left Rotation‐Right	Normal vs. Roussouly 3	−8.3138	0.0000
	Roussouly 1 vs. 2	2.3685	0.0209
	Roussouly 2 vs. 3	−2.0300	0.0466
	Roussouly 2 vs. 4	−2.1879	0.0324
T6–T7‐Right Rotation‐Front	Normal vs. Roussouly 3	4.2332	0.0014
	Roussouly 1 vs. 2	4.8640	0.0005
	Roussouly 1 vs. 3	−4.4625	0.0010
	Roussouly 1 vs. 4	2.5247	0.0282
	Roussouly 2 vs. 3	−4.7976	0.0006
	Roussouly 2 vs. 4	−4.2567	0.0014
	Roussouly 3 vs. 4	5.1094	0.0003
T6–T7‐Right Rotation‐Left	Normal vs. Roussouly 3	12.5881	0.0000
	Roussouly 1 vs. 2	11.0810	0.0000
	Roussouly 1 vs. 3	−22.8473	0.0000
	Roussouly 2 vs. 3	−17.6806	0.0000
	Roussouly 2 vs. 4	−10.3322	0.0000
	Roussouly 3 vs. 4	23.4575	0.0000
T6–T7‐Right Rotation‐Rear	Roussouly 1 vs. 2	3.9228	0.0011
	Roussouly 2 vs. 3	−5.2369	0.0001
	Roussouly 2 vs. 4	−9.0160	0.0000
	Roussouly 3 vs. 4	3.8213	0.0014
T6–T7‐Right Rotation‐Right	Normal vs. Roussouly 3	8.7454	0.0000
	Roussouly 1 vs. 2	3.5413	0.0076
	Roussouly 1 vs. 3	−5.2831	0.0007
	Roussouly 1 vs. 4	4.0151	0.0039
	Roussouly 2 vs. 3	−5.1077	0.0009
	Roussouly 3 vs. 4	6.6522	0.0002
T9–T10‐Right Rotation‐Front	Normal vs. Roussouly 3	−3.7876	0.0010
	Roussouly 1 vs. 2	−3.1189	0.0048
	Roussouly 1 vs. 3	−15.8971	0.0000
	Roussouly 1 vs. 4	−11.3611	0.0000
	Roussouly 2 vs. 3	−5.0090	0.0000
	Roussouly 2 vs. 4	−3.2879	0.0032
	Roussouly 3 vs. 4	12.9879	0.0000
T9–T10‐Right Rotation‐Left	Normal vs. Roussouly 3	4.0171	0.0020
	Roussouly 1 vs. 2	−9.8433	0.0000
	Roussouly 1 vs. 3	−11.8366	0.0000
	Roussouly 1 vs. 4	−9.3808	0.0000
	Roussouly 2 vs. 3	6.4699	0.0000
	Roussouly 2 vs. 4	7.5669	0.0000
	Roussouly 3 vs. 4	9.3808	0.0000
T9–T10‐Right Rotation‐Right	Normal vs. Roussouly 3	−4.0226	0.0020
	Roussouly 1 vs. 2	5.6568	0.0001
	Roussouly 1 vs. 3	−2.3530	0.0383
	Roussouly 2 vs. 3	−10.2651	0.0000
	Roussouly 2 vs. 4	−9.7514	0.0000
	Roussouly 3 vs. 4	7.1059	0.0000
T12–L1‐Right Rotation‐Front	Normal vs. Roussouly 3	−13.7556	0.0000
	Roussouly 1 vs. 2	−15.7129	0.0000
	Roussouly 1 vs. 3	28.9884	0.0000
	Roussouly 1 vs. 4	31.4942	0.0000
	Roussouly 2 vs. 3	23.2511	0.0000
	Roussouly 2 vs. 4	23.3006	0.0000
	Roussouly 3 vs. 4	8.3756	0.0000
T12–L1‐Right Rotation‐Left	Normal vs. Roussouly 3	−5.1051	0.0000
	Roussouly 1 vs. 2	−27.5838	0.0000
	Roussouly 1 vs. 3	12.1434	0.0000
	Roussouly 1 vs. 4	16.7608	0.0000
	Roussouly 2 vs. 3	23.0572	0.0000
	Roussouly 2 vs. 4	26.8025	0.0000
	Roussouly 3 vs. 4	3.7534	0.0012
T12–L1‐Right Rotation‐Rear	Normal vs. Roussouly 3	−2.4348	0.0177
	Roussouly 1 vs. 2	−10.5129	0.0000
	Roussouly 1 vs. 4	3.0006	0.0039
	Roussouly 2 vs. 3	7.9162	0.0000
	Roussouly 2 vs. 4	8.6158	0.0000
	Roussouly 3 vs. 4	3.8806	0.0003
T12–L1‐Right Rotation‐Right	Normal vs. Roussouly 3	4.7607	0.0001
	Roussouly 1 vs. 2	−23.9101	0.0000
	Roussouly 1 vs. 3	13.9374	0.0000
	Roussouly 1 vs. 4	15.3523	0.0000
	Roussouly 2 vs. 3	27.6103	0.0000
	Roussouly 2 vs. 4	31.2869	0.0000
	Roussouly 3 vs. 4	3.2469	0.0039

## References

[1] J. C. Cheng , R. M. Castelein , W. C. Chu , et al., “Adolescent Idiopathic Scoliosis,” Nature Reviews. Disease Primers 1, no. 1 (2015): 15030, 10.1038/nrdp.2015.30. 27188385

[2] A. L. Kuznia , A. K. Hernandez , and L. U. Lee , “Adolescent Idiopathic Scoliosis: Common Questions and Answers,” American Family Physician 101, no. 1 (2020): 19–23. 31894928

[3] US Preventive Services Task Force , D. C. Grossman , S. J. Curry , et al., “Screening for Adolescent Idiopathic Scoliosis: US Preventive Services Task Force Recommendation Statement,” Journal of the American Medical Association 319, no. 2 (2018): 165–172, 10.1001/jama.2017.19342. 29318284

[4] L. G. Lenke , R. R. Betz , J. Harms , et al., “Adolescent Idiopathic Scoliosis: A New Classification to Determine Extent of Spinal Arthrodesis,” Journal of Bone and Joint Surgery. American Volume 83, no. 8 (2001): 1169–1181. 11507125

[5] M. R. Konieczny , H. Senyurt , and R. Krauspe , “Epidemiology of Adolescent Idiopathic Scoliosis,” Journal of Children's Orthopaedics 7, no. 1 (2013): 3–9, 10.1007/s11832-012-0457-4. PMC3566258 24432052

[6] R. B. Graham , P. A. Sugrue , and T. R. Koski , “Adult Degenerative Scoliosis,” Clinical Spine Surgery 29, no. 3 (2016): 95–107, 10.1097/BSD.0000000000000367. 26945131

[7] C. Barrey , J. Jund , O. Noseda , and P. Roussouly , “Sagittal Balance of the Pelvis‐Spine Complex and Lumbar Degenerative Diseases. A Comparative Study About 85 Cases,” European Spine Journal 16, no. 9 (2007): 1459–1467, 10.1007/s00586-006-0294-6. 17211522 PMC2200735

[8] P. Roussouly , S. Gollogly , E. Berthonnaud , and J. Dimnet , “Classification of the Normal Variation in the Sagittal Alignment of the Human Lumbar Spine and Pelvis in the Standing Position,” Spine (Phila Pa 1976) 30, no. 3 (2005): 346–353, 10.1097/01.brs.0000152379.54463.65. 15682018

[9] M. Yu , C. Silvestre , T. Mouton , R. Rachkidi , L. Zeng , and P. Roussouly , “Analysis of the Cervical Spine Sagittal Alignment in Young Idiopathic Scoliosis: A Morphological Classification of 120 Cases,” European Spine Journal 22, no. 11 (2013): 2372–2381, 10.1007/s00586-013-2753-1. 23580056 PMC3886525

[10] S. Fruergaard , M. J. Jain , L. Deveza , et al., “Evaluation of a New Sagittal Classification System in Adolescent Idiopathic Scoliosis,” European Spine Journal 29, no. 4 (2020): 744–753, 10.1007/s00586-019-06241-5. 31802239

[11] Y. P. Charles , P. L. Marchand , N. Tuzin , and J. P. Steib , “Thoracic Kyphosis and Lumbar Lordosis Distribution After Idiopathic Scoliosis Correction Using Posterior Hybrid Versus Screw Instrumentation,” Clinical Spine Surgery 34, no. 6 (2021): E354–E363, 10.1097/BSD.0000000000001171. 33769978

[12] Z. Zhang , Q. Zhou , C. Zhu , L. M. Liu , Y. M. Song , and X. Yang , “Restoring the Ideal Roussouly Sagittal Alignment in Lenke 5 Adolescent Idiopathic Scoliosis Patients: A Method for Decreasing the Risk of Proximal Junctional Kyphosis,” European Spine Journal 33, no. 2 (2023): 695–705, 10.1007/s00586-023-07992-y. 37874394

[13] J. Li , Y. Zhang , Y. Zhang , et al., “Clinical Application of the Roussouly Classification in the Sagittal Balance Reconstruction of 101 Adolescent Idiopathic Scoliosis Patients,” Orthopaedic Surgery 15, no. 1 (2023): 141–151, 10.1111/os.13503. 36398431 PMC9837253

[14] K. E. Pierce , J. M. Mir , P. Dave , et al., “The Incremental Clinical Benefit of Adding Layers of Complexity to the Planning and Execution of Adult Spinal Deformity Corrective Surgery,” Operative Neurosurgery 27, no. 5 (2024): 573–580, 10.1227/ons.0000000000001192. 38771063

[15] S. Galvis , D. Burton , B. Barnds , et al., “The Effect of Scoliotic Deformity on Spine Kinematics in Adolescents,” Scoliosis and Spinal Disorders 11 (2016): 42, 10.1186/s13013-016-0103-x. 27800560 PMC5080732

[16] S. E. Gullbrand , A. Kiapour , C. Barrett , et al., “Restoration of Physiologic Loading After Engineered Disc Implantation Mitigates Immobilization‐Induced Facet Joint and Paraspinal Muscle Degeneration,” Acta Biomaterialia 192 (2024): 128–139, 10.1016/j.actbio.2024.12.014. 39653318 PMC11735281

[17] T. P. Schlösser , M. van Stralen , R. C. Brink , et al., “Three‐Dimensional Characterization of Torsion and Asymmetry of the Intervertebral Discs Versus Vertebral Bodies in Adolescent Idiopathic Scoliosis,” Spine (Phila Pa 1976) 39, no. 19 (2014): E1159–E1166, 10.1097/BRS.0000000000000467. 24921851

[18] Q. Zhang , T. Chon , Y. Zhang , J. S. Baker , and Y. Gu , “Finite Element Analysis of the Lumbar Spine in Adolescent Idiopathic Scoliosis Subjected to Different Loads,” Computers in Biology and Medicine 136 (2021): 104745, 10.1016/j.compbiomed.2021.104745. 34388472

[19] H. Wang , Z. Ma , Z. Wu , et al., “Biomechanical Analysis of Spinal Range of Motion and Intervertebral Disc Loadings in Normal and Adolescent Idiopathic Scoliosis Models,” Frontiers in Bioengineering and Biotechnology 13 (2025): 1473776, 10.3389/fbioe.2025.1473776. 40046810 PMC11880291

[20] J. Y. Hong , K. W. Kim , S. W. Suh , S. Y. Park , and J. H. Yang , “Effect of Coronal Scoliotic Curvature on Sagittal Spinal Shape: Analysis of Parameters in Mature Adolescent Scoliosis Patients,” Clinical Spine Surgery 30, no. 4 (2017): E418–E422, 10.1097/BSD.0000000000000268. 28437347

[21] M. Rasouligandomani , A. Del Arco , F. K. Chemorion , et al., “Dataset of Finite Element Models of Normal and Deformed Thoracolumbar Spine,” Scientific Data 11, no. 1 (2024): 549, 10.1038/s41597-024-03351-8. 38811573 PMC11137096

[22] J. Kok , Y. M. Shcherbakova , T. P. C. Schlösser , et al., “Automatic Generation of Subject‐Specific Finite Element Models of the Spine From Magnetic Resonance Images,” Frontiers in Bioengineering and Biotechnology 11 (2023): 1244291, 10.3389/fbioe.2023.1244291. 37731762 PMC10508183

[23] X. Hu , K. B. Siemionow , and I. H. Lieberman , “Thoracic and Lumbar Vertebrae Morphology in Lenke Type 1 Female Adolescent Idiopathic Scoliosis Patients,” International Journal of Spine Surgery 8 (2014): 30, 10.14444/1030. 25694922 PMC4325490

[24] A. Rohlmann , S. Neller , L. Claes , G. Bergmann , and H. J. Wilke , “Influence of a Follower Load on Intradiscal Pressure and Intersegmental Rotation of the Lumbar Spine,” Spine (Phila Pa 1976) 26, no. 24 (2001): E557–E561, 10.1097/00007632-200112150-00014. 11740371

[25] G. Vaz , P. Roussouly , E. Berthonnaud , and J. Dimnet , “Sagittal Morphology and Equilibrium of Pelvis and Spine,” European Spine Journal 11, no. 1 (2002): 80–87, 10.1007/s005860000224. 11931071 PMC3610486

[26] C. N. Carender , W. Z. Morris , C. Poe‐Kochert , G. H. Thompson , J. P. Son‐Hing , and R. W. Liu , “Low Pelvic Incidence Is Associated With Proximal Junctional Kyphosis in Patients Treated With Growing Rods,” Spine (Phila Pa 1976) 41, no. 9 (2016): 792–797, 10.1097/BRS.0000000000001352. 26656056

[27] S. Ohrt‐Nissen , T. Bari , B. Dahl , and M. Gehrchen , “Sagittal Alignment After Surgical Treatment of Adolescent Idiopathic Scoliosis:Application of the Roussouly Classification,” Spine Deformity 6, no. 5 (2018): 537–544, 10.1016/j.jspd.2018.02.001. 30122389

[28] W. Wang , B. Pei , S. Wu , et al., “Biomechanical Responses of Human Lumbar Spine and Pelvis According to the Roussouly Classification,” PLoS One 17, no. 7 (2022): e0266954, 10.1371/journal.pone.0266954. 35905050 PMC9337691

[29] Z. Kamal , G. Rouhi , N. Arjmand , and S. Adeeb , “A Stability‐Based Model of a Growing Spine With Adolescent Idiopathic Scoliosis: A Combination of Musculoskeletal and Finite Element Approaches,” Medical Engineering & Physics 64 (2019): 46–55, 10.1016/j.medengphy.2018.12.015. 30638786

[30] C. R. D'Andrea , A. F. Samdani , and S. Balasubramanian , “Patient‐Specific Finite Element Modeling of Scoliotic Curve Progression Using Region‐Specific Stress‐Modulated Vertebral Growth,” Spine Deformity 11, no. 3 (2023): 525–534, 10.1007/s43390-022-00636-z. 36593421 PMC10147794

[31] B. A. Walter , C. L. Korecki , D. Purmessur , P. J. Roughley , A. J. Michalek , and J. C. Iatridis , “Complex Loading Affects Intervertebral Disc Mechanics and Biology,” Osteoarthritis and Cartilage 19, no. 8 (2011): 1011–1018, 10.1016/j.joca.2011.04.005. 21549847 PMC3138834

[32] A. Sebaaly , P. Grobost , L. Mallam , and P. Roussouly , “Description of the Sagittal Alignment of the Degenerative Human Spine,” European Spine Journal 27, no. 2 (2018): 489–496, 10.1007/s00586-017-5404-0. 29177554

[33] Z. Zhang , T. Wang , H. Bian , et al., “A Novel Approach to Predict the Location and Fatigue Life of Intervertebral Disc Degeneration,” Bioengineering (Basel) 12, no. 4 (2025): 423, 10.3390/bioengineering12040423. 40281783 PMC12025289

[34] N. Buchweitz , Y. Sun , J. Kelley , et al., “Characterizing the Baseline Regional Biphasic Mechanical Properties of Cervical Intervertebral Discs,” Annals of Biomedical Engineering 53 (2025): 2333–2345, 10.1007/s10439-025-03759-2. 40399746 PMC12391200

